# Artificial Intelligence-Powered Surgical Consent: Patient Insights

**DOI:** 10.7759/cureus.68134

**Published:** 2024-08-29

**Authors:** Alex Teasdale, Laura Mills, Rhodri Costello

**Affiliations:** 1 Ear Nose Throat, Morriston Hospital, Swansea, GBR; 2 General Practice, Dyfed Road Surgery, Swansea, GBR

**Keywords:** consent process, ai in healthcare, patient autonomy, data privacy, medical ethics, healthcare innovation, chatgpt, patient engagement, surgical consent, artificial intelligence

## Abstract

Introduction

The integration of artificial intelligence (AI) in healthcare has revolutionized patient interactions and service delivery. AI's role extends from supporting clinical diagnostics and enhancing operational efficiencies to potentially improving informed consent processes in surgical settings. This study investigates the application of AI, particularly large language models like OpenAI's ChatGPT, in facilitating surgical consent, focusing on patient understanding, satisfaction, and trust.

Methods

We employed a mixed-methods approach involving 86 participants, including laypeople and medical staff, who engaged in a simulated AI-driven consent process for a tonsillectomy. Participants interacted with ChatGPT-4, which provided detailed procedure explanations, risks, and benefits. Post-interaction, participants completed a survey assessing their experience through quantitative and qualitative measures.

Results

Participants had a cautiously optimistic response to AI in the surgical consent process. Notably, 71% felt adequately informed, 86% found the information clear, and 71% felt they could make informed decisions. Overall, 71% were satisfied, 57% felt respected and confident, and 57% would recommend it, indicating areas needing refinement. However, concerns about data privacy and the lack of personal interaction were significant, with only 42% reassured about the security of their data. The standardization of information provided by AI was appreciated for potentially reducing human error, but the absence of empathetic human interaction was noted as a drawback.

Discussion

While AI shows promise in enhancing the consistency and comprehensiveness of information delivered during the consent process, significant challenges remain. These include addressing data privacy concerns and bridging the gap in personal interaction. The potential for AI to misinform due to system "hallucinations" or inherent biases also needs consideration. Future research should focus on refining AI interactions to support more nuanced and empathetic engagements, ensuring that AI supplements rather than replacing human elements in healthcare.

Conclusion

The integration of AI into surgical consent processes could standardize and potentially improve the delivery of information but must be balanced with efforts to maintain the critical human elements of care. Collaborative efforts between developers, clinicians, and ethicists are essential to optimize AI use, ensuring it complements the traditional consent process while enhancing patient satisfaction and trust.

## Introduction

Although the impact of artificial intelligence (AI) in healthcare is yet to be fully realized, in recent years, the role of AI in transforming various industries has been profound. Despite ethical and medico-legal concerns as well as uncertainty regarding workforce utilization, its integration into various healthcare applications promises transformative changes across the spectrum of medical practice, particularly in patient interaction and service delivery [1]. The application of AI in healthcare is already extensive. It supports clinical processes by aiding in disease diagnosis, forecasting patient outcomes, and tailoring treatment approaches [2]. Within hospital management, AI enhances operational efficiencies, simplifies administrative procedures, and optimizes patient scheduling and flow [3]. In medical diagnostics, AI improves the precision and speed of image interpretation in areas like radiology and pathology [4]. Furthermore, AI significantly influences patient care through its integration into remote monitoring, telemedicine, and virtual support, revolutionizing the interaction between patients and healthcare providers [5].

AI is everywhere, and one additional use may be to obtain informed consent from patients in preparation for surgical procedures, a critical element of ethical and safe healthcare practice. Barriers exist within the current consenting process, which may cause anxiety and stress and affect the patient's ability to comprehend and process the information being given, especially when overloaded with information in a short timeframe. Patients must understand the implications of the procedures they are about to undergo, and traditional consent processes, while thorough, often vary significantly in how information is conveyed, depending on the clinician’s style and the setting. In the National Health Service (NHS), it is standard for parts of this consent process to be handled by team members other than the clinician who will perform the procedure [6]. The General Medical Council (GMC) guidelines around consent delegation aim to ensure that the individual taking consent is competent, appropriately trained, and has sufficient knowledge of the procedure including the risks and benefits as well as alternative options [7]. Therefore, the task of obtaining consent often falls to a junior doctor, qualified medical professionals who, despite having earned their medical degrees, are still undergoing clinical training under the oversight of more experienced clinicians [8]. Their level of experience as a doctor may range from a few years to a decade or more. The aim of delegation is to optimize the clinical workflow and ensure focused time for patients to make informed decisions while additionally ensuring that the junior doctors acquire the necessary knowledge about the procedures and the legal aspects of the consent process [7].

Despite the stringent legal and ethical guidelines established by the GMC, these standards are often not met in practice. Many junior doctors handling the consent process are not adequately trained or knowledgeable about the procedures [8,9]. There is also inconsistency among surgeons about the extent of information that should be disclosed before surgery, with different surgeons offering different information to the patients [10].

Ideally, there should be ample time for detailed discussions with patients. While bigger operations are usually discussed at length in a clinic, consent for smaller procedures is commonly sought either on the day of the surgery or just moments before the surgery, limiting the time available for patients to make informed decisions and potentially affecting the voluntariness of their consent. The effect of this is exaggerated when doctors are pressured by other clinical responsibilities and time constraints [11]. These shortcomings in the consent process not only threaten the validity of the informed consent obtained but also risk damaging the trust between the public and medical practitioners, as well as the legal safeguards for clinicians.

The question then arises: Could the consent process be delegated to AI? There is growing enthusiasm about the potential advantages of integrating generative AI, including the emergence of large language models (LLMs) like OpenAI's ChatGPT into the consenting process. Generative Pre-trained Transformer programs, such as ChatGPT, offer revolutionary processing of natural language in clinical notes, allowing more accurate extraction of patient information. These models are capable of handling tasks traditionally exclusive to humans and have exhibited strong performance in medical knowledge assessments even without being trained specifically on medical data [12].

By standardizing this process through AI, there is potential not only to maintain consistency in the information delivered but also to enhance patient engagement and understanding. Studies have shown that patients like the personalized element of AI consenting and find it engaging and user-friendly [13]. However, many questions remain unanswered regarding public perception, trust, and the impact on the doctor-patient relationship [14]. This research aims to explore the viability and efficacy of using AI, particularly the AI system ChatGPT, as a part of the surgical consent process. We assess patient perceptions, focusing on their satisfaction, understanding, and trust in the process, while identifying areas that could benefit from further improvement.

The advent of AI technology presents an opportunity to mitigate these issues by providing a consistent, detailed dialogue tailored to the patient’s needs. The implementation of AI in this context is not intended to replace human interaction but to support it by providing a standardized baseline of information, ensuring that all patients receive the same level of detail in their briefing. This study explores the public's beliefs in the hypothesis that AI can enhance the surgical consenting process, making it more consistent and perhaps even more comprehensive than conventional methods.

## Materials and methods

This study aimed to evaluate the effectiveness and reception of AI-powered surgical consent using a mixed-methods approach, combining both quantitative and qualitative data to provide a comprehensive assessment of the AI system's impact. The study was conducted for three months and involved a total of 86 participants drawn from a diverse pool, including laypeople, nurses, and surgical staff, simulating a real-world healthcare setting. Participants were recruited through advertisements within a large teaching hospital and the local community.

Inclusion criteria required participants to be 18 years or older and proficient in English to ensure effective communication with the AI system. Participants were chosen to represent a range of perspectives and experiences, including those unfamiliar with medical procedures, nurses with intermediate knowledge, and surgical staff with extensive experience. This variety aimed to capture a holistic view of AI's impact on the consent process. Potential participants were screened for eligibility and consented to participate in the study after being informed about its purpose and procedures.

Participants were briefed about the study protocol before engaging with the AI system. Each participant was instructed to assume the role of a mock surgical patient scheduled for a tonsillectomy. The interaction with ChatGPT-4 was designed to replicate a typical preoperative consultation, where patients receive information about the surgical procedure. Participants were encouraged to ask questions and engage in a dialogue with the AI to address any concerns or clarify doubts. The AI interaction lasted approximately 15-20 minutes, allowing sufficient time for participants to absorb information and ask follow-up questions. An example conversation can be found in Appendix 1.

After the AI interaction, participants completed a comprehensive survey designed to capture both quantitative and qualitative feedback. The study was designed by the corresponding author, who was trained by the local health board in data collection, in the form of an online questionnaire created using Google Forms. It was provided to the participants via email or on a separate computer for those who did not wish to provide an email address. It was completed anonymously. The questionnaire consisted of several sections, including demographic information, satisfaction ratings, and open-ended questions. Quantitative measures included Likert-scale items assessing participant satisfaction with the AI interaction, perceived adequacy of the information provided, and confidence in the AI-assisted consent process. These items were designed to quantify participant perceptions of the AI's effectiveness and their overall experience.

Qualitative questions aimed to elicit detailed feedback about participants' experiences, capturing insights into their understanding of the information, perceived benefits, and potential drawbacks of AI-assisted consent. Open-ended questions invited participants to share their thoughts via free text boxes, on the strengths and weaknesses of the AI system, its impact on their decision-making process, and any concerns they had about AI's role in healthcare.

The study was approved by the Institutional Review Board (IRB) of the participating institution, ensuring that all ethical guidelines and standards were adhered to throughout the research process. Participants were informed about the study's purpose, procedures, potential risks, and benefits before providing written informed consent. Confidentiality was maintained by assigning unique identifiers to each participant and securely storing data in password-protected databases. Participants were assured that their participation was voluntary and that they could withdraw from the study at any time without any consequences.

## Results

**Figure 1:**
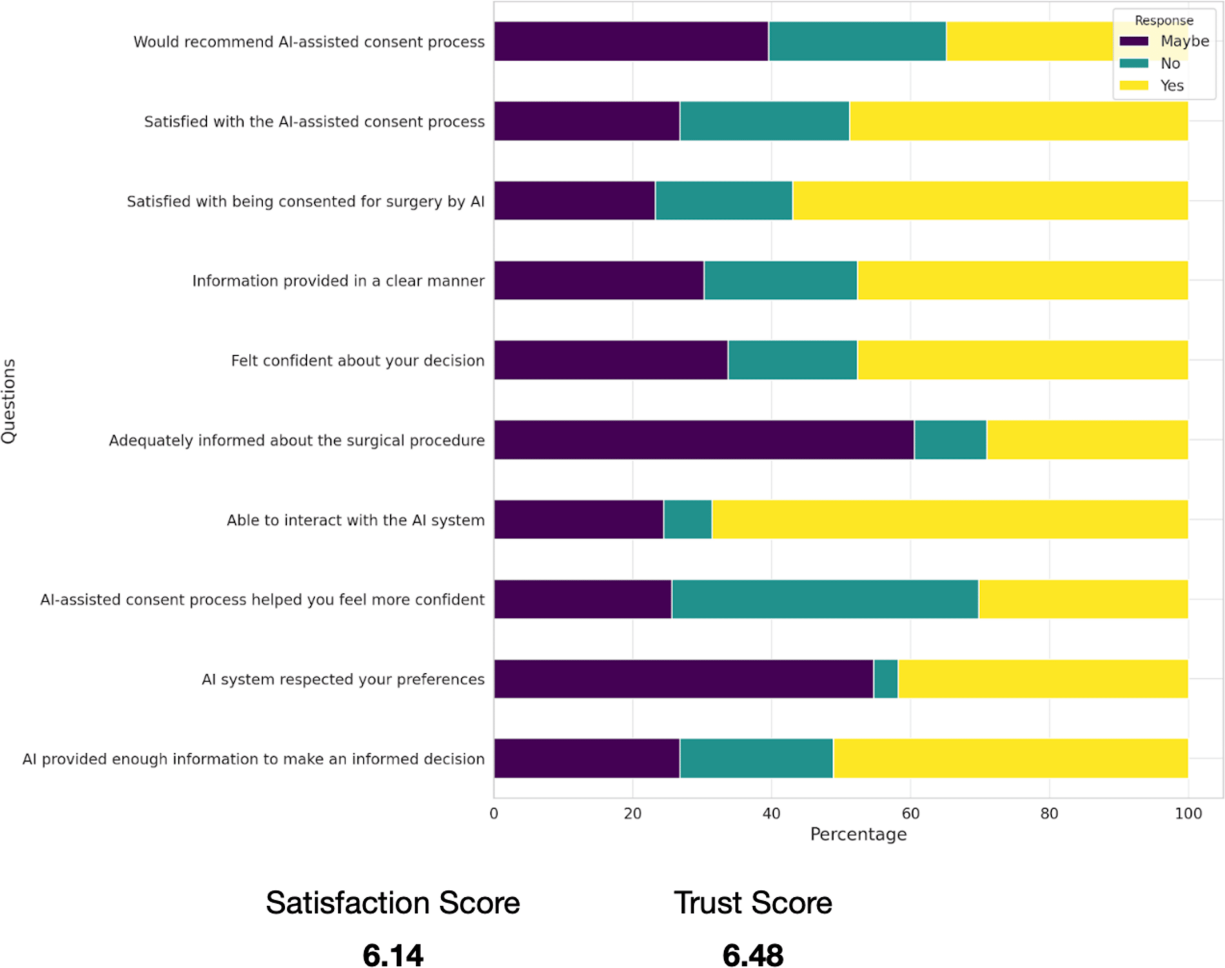
Graphical representation of participants' responses to the post-consent survey

The incorporation of AI into the surgical consent process elicited a cautiously optimistic reaction from participants, as illustrated in Figure 1. In terms of quantitative feedback, the participants reported varying levels of satisfaction with different aspects of the AI system. Notably, 71% of the participants felt adequately informed about the surgical procedure, while 86% found the information provided by the AI system to be clear. Interaction with the AI system was satisfactory for 86% of the participants, and 71% felt that the AI system provided enough information to make an informed decision.

Regarding the overall satisfaction with the AI-assisted consent process, 71% of participants expressed satisfaction. Confidence in the AI system's respect for their preferences was reported by 57% of participants. Furthermore, 57% of participants felt that the AI-assisted consent process helped them feel more confident about their decision, and 71% felt confident about their decision after using the AI system. Lastly, 57% of participants indicated they would recommend the AI-assisted consent process to others. These statistics not only indicate a generally positive reception but also highlight specific areas where AI systems require further refinement to achieve wider acceptance.

**Figure 2:**
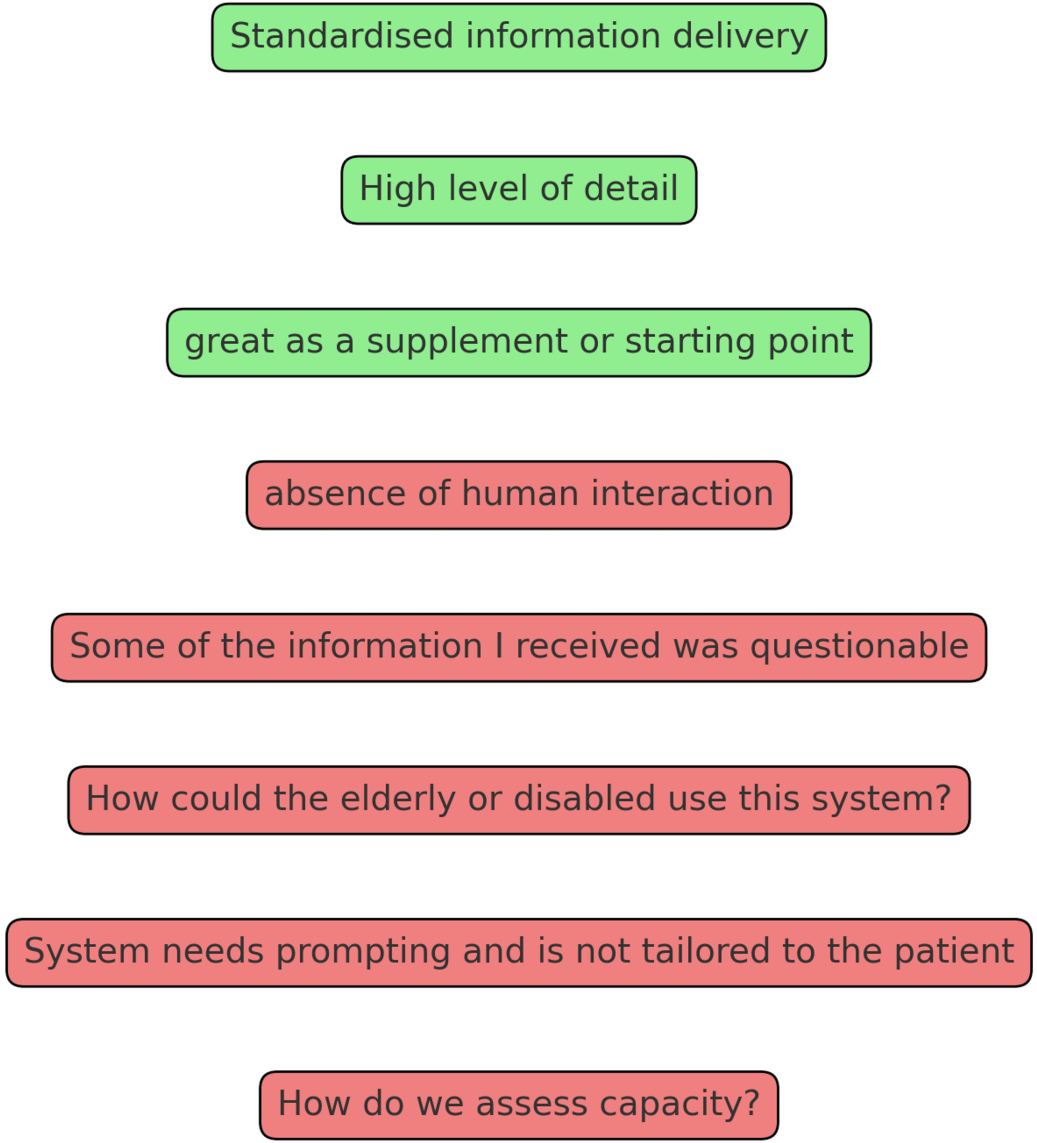
Selected example quotes provided by participants

From a qualitative perspective, the primary benefits included the standardization of information, which participants believed could diminish human error and variability in the information delivered, as shown in Figure 2. The efficiency of the AI-driven process was also appreciated, as many participants recognized the potential for time savings before surgery. The detail provided by the AI was considered superior compared to some prior experiences with human healthcare providers. Nonetheless, notable disadvantages were highlighted, especially the absence of a personal touch. Participants expressed a lack of the reassurance and empathy that are typically afforded by human interactions and deemed essential in clinical environments. Additionally, there were concerns about the AI system's inability to assess the user's comprehension in real-time and adjust the provided information based on the user's needs and understanding.

## Discussion

AI has shown huge potential in improving diagnostic accuracy, creating personalized treatments, and enhancing the efficacy of the healthcare system. However, the widespread use of AI in other industries has attracted criticism, especially about data privacy and ethical concerns, a finding supported by this study [15-17]. Significant skepticism was observed among participants about how their data was being used, creating an overriding lack of trust. This lack of confidence likely stems from recent examples of data breaches and the common knowledge that programs such as Google’s DeepMind have access to millions of identifiable patient records from the NHS [18]. Attempts to maximize compliance with the Health Insurance Portability and Accountability Act (HIPAA) and General Data Protection Regulation (GDPR) have been made by using "Prompt-Induced Sanitization" to reduce input Regurgitation [19]. Despite this, there is a significant worry that AI systems could be susceptible to cyberattacks [20].

AI technologies combine several advanced techniques: They utilize artificial neural networks capable of learning from extensive data sets, known as deep neural networks (DNN); they process and understand human language using natural language processing (NLP), and they enhance their performance autonomously over time without direct human guidance. These capabilities enable AI to handle complex tasks with increasing efficiency and accuracy. However, they also require the system to absorb huge volumes of personal data, and patients do not necessarily understand how their data is used [17]. The handling of personal data is not specific to AI; it already exists with a large variety of electronic patient record systems, which are regulated by the GDPR. This regulation must be applied to AI systems involved in obtaining consent.

The failure to trust the system is exacerbated by the lack of "human" interaction, although some studies have found that AI can effectively simulate consent conversations that are sensitive and empathetic [21]. However, despite terms like "learn," "think," and "decide" often being applied to describe AI behaviors, these systems do not possess sentience. They function similarly to standard computer programs, processing inputs to produce outputs [22]. Studies have found that obtaining consent in written form should not be considered sufficient as patients do not find it reassuring. They often lack full awareness of their health choices, so orally delivered information is significantly better for the patient's needs [23].

Whether a patient is interacting with an AI system or a medical professional, ensuring the accuracy of the information provided is essential. AI is an effective tool for making medical information, such as leaflets, more readable. However, in these cases, it is not generating new information, but rather rearranging pre-existing material [24,25].

Participants suggested that a lack of trust is in part due to the risk of misinformation, largely due to the obscure nature of AI systems. It may be challenging, or even impossible, to trace the origins of specific outputs from an AI system, which will occasionally produce convincing but inaccurate statements, known as "hallucinations" [26]. The combination of random "hallucinations" and the inherent biases in the training data could alter the information provided and potentially lead to patients making important decisions based on incorrect information, compromising the legitimacy of patient consent [27]. However, it should be noted that human-managed consent processes are also susceptible to inaccuracies and biases [28]. A notable benefit of using an AI system is the ability to record and verify consent discussions, allowing any conveyed information to be audited and validated.

Reviewing the conversations allows the primary clinician to maintain the ultimate responsibility outlined in the GMC guidelines. The concept of ownership or responsibility for AI is contentious. Some would argue that as AI lacks autonomy, it also lacks the "moral agency" that a human might have; therefore, it is the responsibility of the primary clinician to review the information provided [29,30]. Consequently, it is key that both the patients and the surgeon trust the AI system. A recent study investigating the views of medical professionals suggests that the majority believe AI will positively impact healthcare and would be open to including it in their practice, with regard to decision-making, patient support, literature reviews, and research assistance. However, significant concerns remain about the credibility of the information provided [31].

Clinicians must also trust that the patient can read, absorb, and make an informed decision based on the given information, which is not currently assessed by an AI model. When presented with large volumes of complex information, such as the risks of a procedure, patients are known to attempt to skip to the end without sufficiently understanding the information provided, just as they might with "terms and conditions" [13].

Limitations

The small sample size of 86 participants, primarily consisting of laypeople, nurses, and surgical staff, may not accurately represent the broader population. Additionally, the study's simulated environment does not capture the dynamics of real-world surgical settings and the realistic decision-making that a genuine tonsillectomy would offer. A lack of a control group using traditional consent methods limits the ability to compare AI-assisted consent to existing practices. Furthermore, the study's focus on a single procedure, tonsillectomy, may be slightly restricting when applying this to more complicated surgery.

Future studies should address these limitations by conducting larger, more diverse studies in real clinical settings, incorporating control groups, and evaluating AI performance across various procedures. Moreover, longitudinal studies could provide insights into the long-term impact of AI-assisted consent on patient outcomes. Collaborating with AI developers, healthcare providers, and ethicists can help refine AI systems to align with clinical best practices, addressing ethical concerns and enhancing human-AI interaction in the consent process. These improvements will provide robust evidence of AI's role in surgical consent, ensuring ethical standards and patient needs are met.

## Conclusions

The study findings illuminate the complex balance between technological innovation and human-centered care. While AI can enhance the consistency and efficiency of surgical consent, it lacks the nuanced understanding and empathetic engagement that human practitioners offer. This gap underscores the necessity for a hybrid approach, where AI supports rather than replaces human interaction. Improving AI systems to recognize and adapt to non-verbal cues or to solicit feedback actively during the consent process could address some of these concerns. This research demonstrates a significant interest in and potential for incorporating AI into the surgical consent process, with an emphasis on its ability to standardize and detail information delivery. Some studies have suggested that AI is capable of accurately predicting risk for perioperative and postoperative complications based on large audit data. Therefore, AI could provide personalized recommendations for operative management and supplement the informed consent process. However, the integration of such technology should not overlook the value of human touch, which plays a critical role in patient comfort and satisfaction.

Future research should aim to refine AI capabilities, focusing on enhancing interactive features and addressing privacy concerns, to better support both patients and healthcare providers. The path forward involves rigorous testing and development of AI systems to handle more complex interactions, such as assessing comprehension and adapting explanations accordingly. Collaborative efforts between AI developers, clinicians, and ethicists are essential to create a system that respects patient privacy, enhances understanding, and maintains the human elements critical to the consent process. Further studies with larger, more diverse populations can provide deeper insights and help refine the use of AI in clinical settings, ensuring that it meets the broad needs of patients across different demographics.

## Figures and Tables

**Figure 3:**
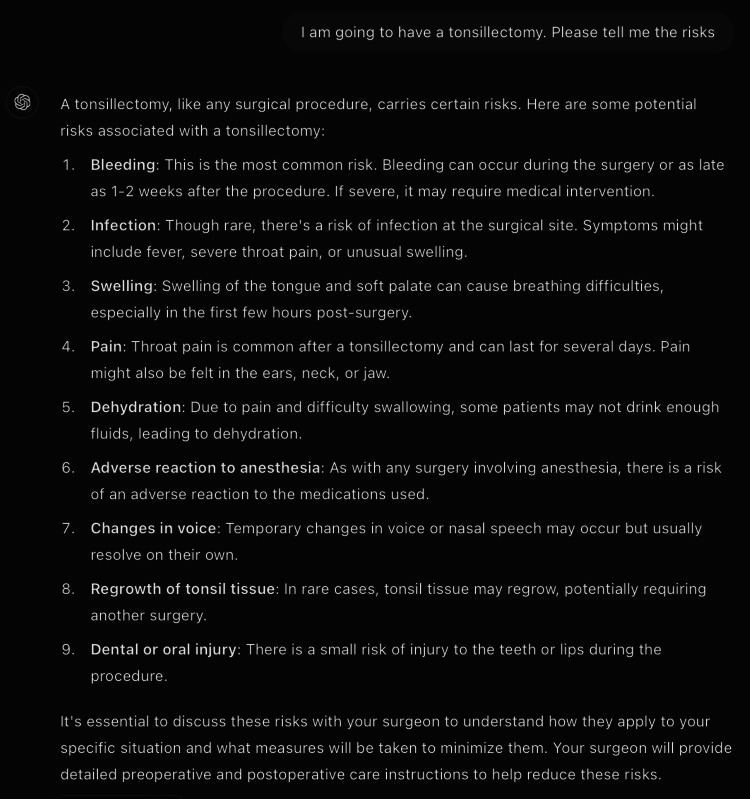
The explanation of risks for a tonsillectomy as provided by ChatGPT-4o

**Figure 4:**
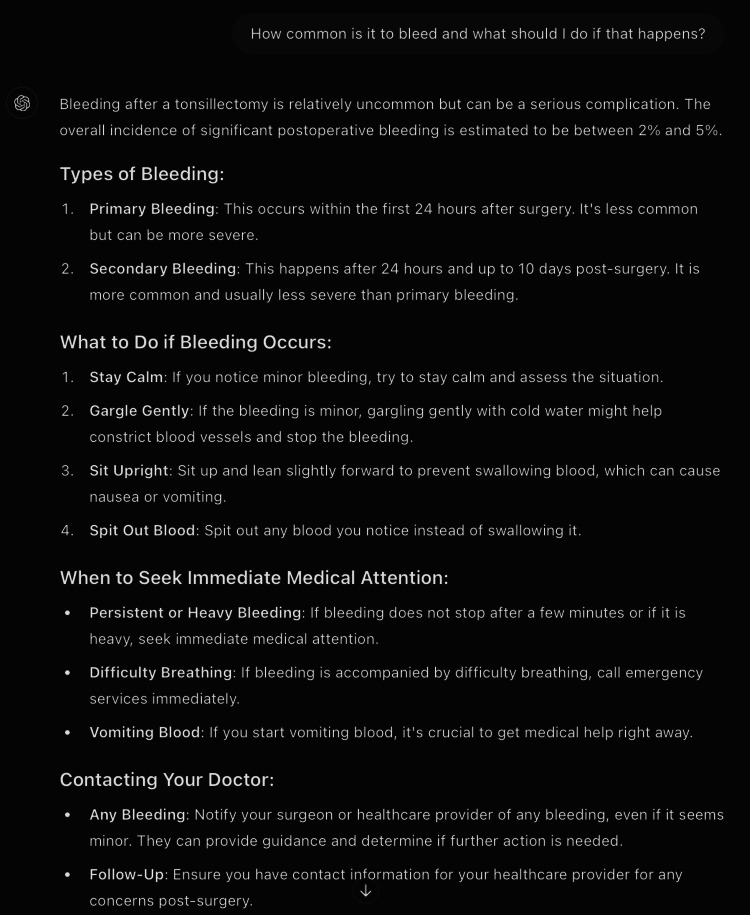
An explanation of bleeding risk as provided by ChatGPT-4o

**Figure 5:**
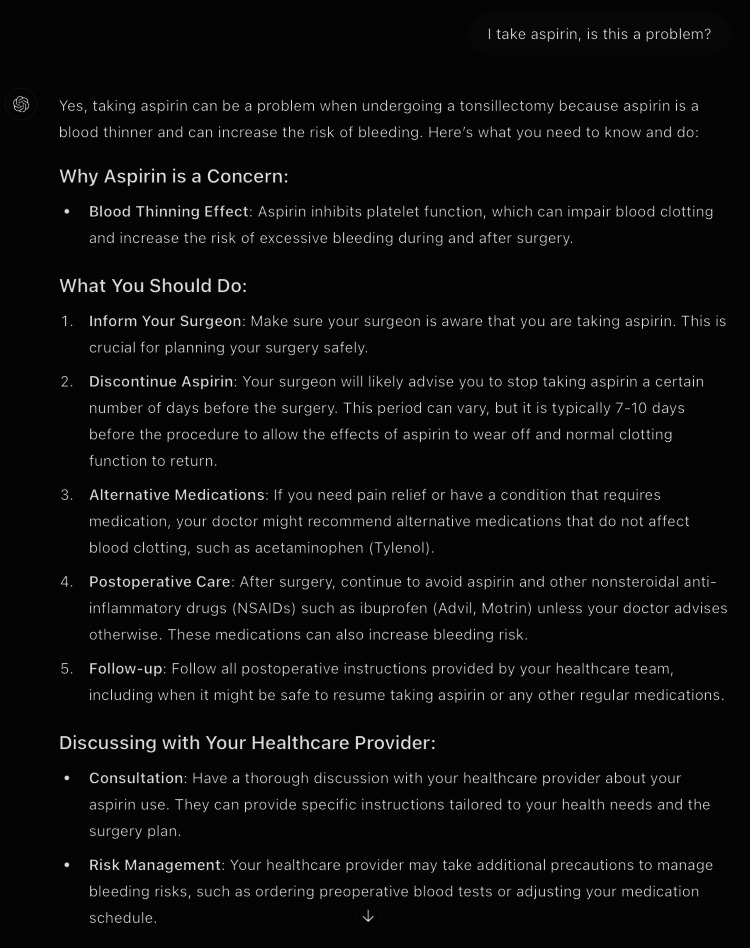
An explanation of the risk of having a tonsillectomy while taking an anticoagulant (aspirin) as described by ChatGPT-4o

## Appendices

Appendix 1: Snapshots of an example conversation between a participant and ChatGPT-4
